# CsPbBr_3_ Perovskite Nanocrystals in P3HT:PCBM Hybrid Photodetectors: Spectral Enhancement and Evidence for Photoinduced Energy Transfer

**DOI:** 10.3390/polym18070808

**Published:** 2026-03-26

**Authors:** Fernando Rodríguez-Mas, José Luis Alonso Serrano, Pablo Corral González, Abraham Ruiz Gómez, Juan Carlos Ferrer Millán

**Affiliations:** 1 Communications Engineering Department, Miguel Hernandez University, 03202 Elche, Spain; pcorral@umh.es (P.C.G.); abraham.ruizg@umh.es (A.R.G.); 2 University Institute for Engineering Research, Miguel Hernandez University, 03202 Elche, Spain; j.l.alonso@umh.es (J.L.A.S.); jc.ferrer@umh.es (J.C.F.M.)

**Keywords:** CsPbBr3 perovskite nanoparticles, organic photodetectors, self-powered devices, P3HT:PCBM, hybrid optoelectronic devices, visible light communication

## Abstract

We report the enhancement of organic photodetector (OPD) performance through the incorporation of CsPbBr_3_ perovskite nanocrystals (PNCs) into P3HT:PCBM devices. The optimized device (HPD_01) exhibits a maximum responsivity of 0.083 A/W and a specific detectivity of ~4.7 × 10^10^ Jones, and a minimum NEP of 5.2 × 10^−12^ W·Hz^−1/2^ at the self-powered operating point (V ≈ 0 V), outperforming the nanoparticle-free reference. Frequency- and distance-dependent measurements under visible light communication conditions demonstrate that the optimized device maintains strong signal detection up to 1 MHz and at distances exceeding 15 cm. Notably, the external quantum efficiency spectra reveal an additional contribution in the 450–575 nm range, which is absent in the reference device. This enhancement is consistent with a radiative absorption–reemission energy-transfer mechanism, supported by quantitative spectral overlap analysis showing that 99.5% of the PNC photoluminescence falls within the 450–575 nm EQE enhancement window and that the maximum differential EQE gain occurs at 519 nm—only 2 nm from the PNC emission peak. Our results suggest that controlled PNC incorporation enables efficient optical energy coupling, leading to high-sensitivity, fast-response OPDs suitable for optical communication applications.

## 1. Introduction

**Figure 1:**
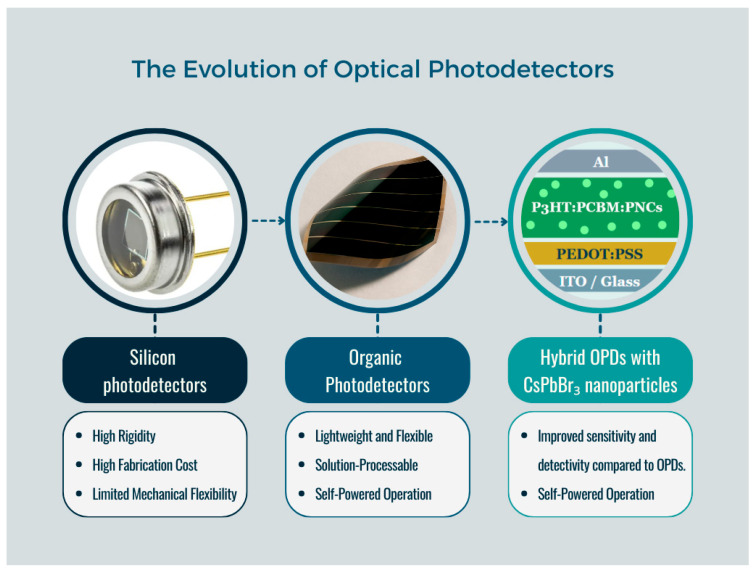
The evolution of optical photodetectors.

The development of advanced photonic and optoelectronic materials is a cornerstone of modern technology [1], enabling innovations in areas such as optical communication [2], biomedical imaging [3], and solar energy conversion [4]. The past decade has seen rapid growth in technologies based on light–matter interactions, driving the search for materials that combine high optical sensitivity [5], structural stability [6], and compatibility with scalable fabrication processes [7]. In this context, organic photodetectors (OPDs) have emerged as a versatile, cost-effective platform for converting light into electrical signals [8]. Their key advantages include mechanical flexibility, solution-processable fabrication, and integration onto transparent substrates [9,10,11] (Figure 1).

In contrast to conventional silicon-based detectors, OPDs can operate in self-powered configurations [12]. This functionality, enabled by p–n diode or donor–acceptor heterojunction architectures [13], allows the development of energy-efficient devices suitable for portable or wireless applications, including autonomous optical sensors [14] and visible light communication (VLC) systems [15]. Moreover, the organic semiconducting active layer permits tuning of optical and electronic properties through the rational choice of donor and acceptor components [16].

The poly(3-hexylthiophene) (P3HT) and [6,6]-phenyl-C_61_-butyric acid methyl ester (PCBM) system (P3HT as donor and PCBM as acceptor) is a well-established model for studying organic photoelectric conversion [17]. Its bicontinuous morphology promotes exciton dissociation and efficient charge transport [18], making it an ideal platform for exploring hybrid strategies. However, devices based solely on P3HT:PCBM are limited by low charge carrier mobility and significant non-radiative recombination [19,20]. To overcome these challenges, hybridization with inorganic nanomaterials—such as perovskite nanocrystals (PNCs)—offers a promising approach [21]. PNCs can enhance light absorption, charge transport, and excitonic energy transfer through quantum confinement and resonance effects [22,23,24,25,26,27]. In particular, CsPbBr_3_ perovskites provide high optical absorption, strong green photoluminescence, and remarkable thermal and moisture stability [23,24,25], enabling the formation of hybrid heterojunctions that can support self-powered photodetector operation. This hybrid strategy represents a promising frontier in advanced optical materials, aligned with trends toward device miniaturization and energy-efficient photonics.

### 1.1. Current Limitations of Organic Photodetectors

Despite significant progress, OPDs still face structural, optical, and electronic limitations that restrict their performance relative to conventional inorganic detectors [14]. Their operation relies on converting incident photons into free charge carriers via electron–hole pair excitation in organic semiconductors [28]. However, the weak molecular bonding, low charge mobility, and strongly bound excitons of these materials hinder efficient charge separation and transport [29].

**Figure 2:**
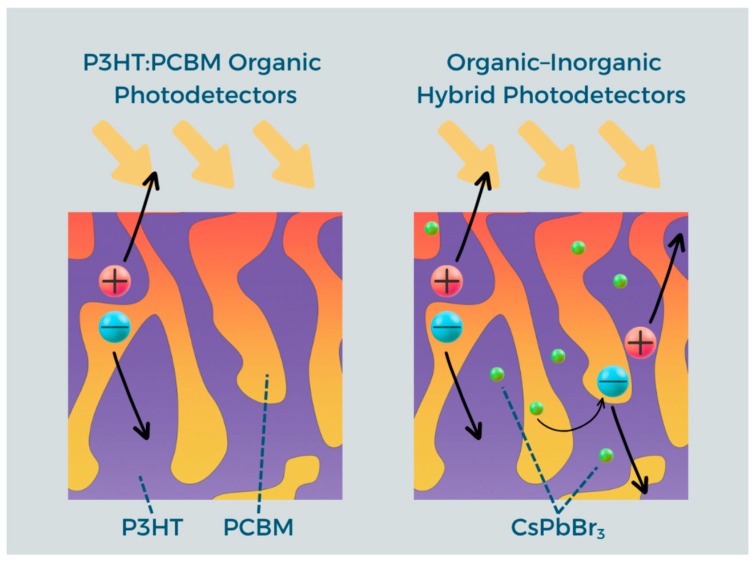
A conceptual comparative scheme between a P3HT:PCBM organic photodetector and an organic–inorganic hybrid photodetector.

Environmental degradation further limits device stability: humidity, oxygen, and UV radiation can induce photochemical and oxidative reactions in both the active layer and metallic contacts [30,31]. Encapsulation and stabilizing dopants can mitigate these effects but add complexity and cost [32]. OPDs also exhibit dynamic limitations, as charge accumulation at the donor–acceptor interface and parasitic capacitance reduce response speed [33,34], restricting their use in high-speed applications such as VLC [35]. These challenges highlight the need for hybrid strategies, with the incorporation of inorganic nanoparticles—particularly CsPbBr_3_ perovskite nanocrystals—offering a promising route (Figure 2).

### 1.2. Introduction of the Concept of Hybridization with CsPbBr_3_ Perovskites

Incorporating inorganic nanomaterials into the active layer of self-powered OPDs has shown notable advances, with metallic nanoparticles (Au, Ag) [36] enhancing optical absorption through plasmonic effects, and semiconductor oxides (ZnO, TiO_2_) improving electron mobility and device stability [37]. However, both approaches can suffer from interfacial recombination and integration challenges. In contrast, PNCs offer superior energetic alignment and morphological compatibility, making them an effective alternative for hybrid OPD architectures [38]. Metal halide perovskites (ABX_3_, where A = Cs^+^, MA^+^ or FA^+^; B = Pb^2+^ or Sn^2+^; X = Cl^−^, Br^−^ or I^−^) exhibit high absorption coefficients (>105 cm^−1^), elevated electron mobility, and tunable bandgaps through nanoscale crystal control [39]. These properties have enabled applications ranging from solar cells and LEDs to radiation detectors and tunable lasers [40,41,42,43]. Despite these advances, most studies focus on static photovoltaic parameters (Power Conversion Efficiency (PCE), Open-Circuit Voltage (V_OC_) or Short-Circuit Current Density (J_SC_)), while dynamic performance metrics—such as electronic noise, specific detectivity (D*), noise-equivalent power (NEP), and signal-to-noise ratio (SNR, defined in Section 3.3 as the ratio of net photocurrent to noise current spectral density)—remain underexplored. Systematic analysis of NEP, SNR, and D* across different PNC concentrations provides a quantitative strategy to optimize hybridization, enhancing both sensitivity for low-intensity light detection and suitability for high-speed optical communication [43].

This work investigates how varying concentrations of CsPbBr_3_ PNCs affect the optical, electrical, and dynamic performance of self-powered OPDs. We employ a comprehensive set of characterization techniques—including J–V measurements, absorption and photoluminescence spectroscopy, electronic noise analysis, responsivity, detectivity, and frequency response—to correlate active-layer composition with device performance. This multidimensional approach provides insights into the role of PNCs in modulating light–matter interactions, guiding the rational design of next-generation hybrid photodetectors.

## 2. Materials and Methods

### 2.1. Materials

Lead(II) bromide (PbBr_2_, ≥99%), cesium bromide (CsBr, ≥99%), anhydrous dimethyl sulfoxide (DMSO) Poly(3,4-ethylenedioxythiophene) polystyrene sulfonate (PEDOT:PSS), P3HT, PCBM, toluene, acetone, isopropanol, and deionized water were used as received, without any further purification. All reagents were handled under a controlled ambient atmosphere and stored under dry conditions to prevent moisture absorption. All reagents were obtained from Sigma-Aldrich (St. Louis, MO, USA).

### 2.2. Synthesis of Perovskite Nanoparticles (CsPbBr_3_)

The perovskite nanoparticles were synthesized using a rapid-injection method into an antisolvent medium. First, 36.7 mg of PbBr_2_ were dissolved in 1.0 mL of anhydrous DMSO under stirring at 50 °C until a clear solution was obtained. Subsequently, 63.8 mg of CsBr were added, maintaining a Cs:Pb molar ratio of 3:1. This precursor excess is an intentional synthetic strategy commonly employed in rapid-injection protocols to favor nucleation of the perovskite phase, suppress non-perovskite secondary phases and improve colloidal stability of the resulting nanocrystals. Stirring was continued until complete dissolution of the precursors [44,45]. In parallel, 10 mL of cold toluene was prepared to serve as the antisolvent. The hot precursor solution was rapidly injected into the toluene under stirring, and the mixture was maintained for approximately 30 s. During this process, CsPbBr_3_ perovskite nanoparticles were formed. The synthesized nanoparticles were recovered by decantation and centrifugation. The precipitate was separated from the supernatant. This procedure removed solvent residues and unreacted salts, ensuring good colloidal dispersibility.

### 2.3. Fabrication of Hybrid Photodetectors

The hybrid photodetector (HPD) devices were fabricated with the structure ITO/PEDOT:PSS/P3HT:PCBM: PNCs/Al, where PNCs were incorporated into the active layer composed of the P3HT:PCBM blend. Glass substrates coated with indium tin oxide (ITO) were used due to their high optical transparency and electrical conductivity, which are essential for their function as the device anode. The substrates were cleaned via a sequential ultrasonic process in acetone, isopropanol, and deionized water for 15 min each. Subsequently, they were dried under a nitrogen (N2) flow and treated with ozone for 5 min to improve surface wettability and enhance adhesion of the subsequent layers. The PEDOT:PSS layer was deposited onto the ITO via spin-coating at 6000 rpm for 60 s, forming a thin and uniform film that acts as a hole transport layer and surface planarization layer. The coated substrates were then subjected to thermal annealing at 150 °C for 10 min to remove residual solvent and improve the film conductivity. The active layer was prepared by dissolving the donor polymer P3HT and the acceptor PCBM in a mass ratio of 1:0.8 using the organic solvent chlorobenzene. Various amounts of CsPbBr_3_ perovskite nanoparticles were added to this mixture to evaluate their effect on the optoelectronic properties of the device. The proportions used for each device are detailed in Table 1, varying the PNC concentration from 0 mg (reference) to 8.333 mg, corresponding to P3HT:PCBM: PNC ratios of 1:0.8:x, where x = 0, 0.5, 1.0, 2.0, 5.0, and 10.0, respectively. The total volume of solution deposited was 75 μL per device. The nominal PNC content ranges from 0 to 29.40 wt% relative to the total solid content (P3HT + PCBM + PNCs).

The active layer was deposited via spin-coating at 3000 rpm for 60 s, producing a uniform film. Subsequently, the samples underwent thermal annealing at 150 °C for 10 min to promote P3HT crystallization, enhance the blending of components, and ensure proper dispersion of the nanoparticles within the organic matrix. Finally, a metallic aluminum (Al) layer of approximately 100 nm thickness was deposited via high-vacuum thermal evaporation (pressure below 10^−6^ mbar). This electrode functions as the device cathode. The film thickness was monitored in real time using a quartz crystal microbalance to ensure reproducibility across different devices. Only one representative device per composition was characterized in the present study; the process controls described above were applied to all devices to minimize inter-device variability. After cathode deposition, the devices were encapsulated to protect them from atmospheric moisture and oxygen, thereby preserving their stability during optoelectronic measurements. No light soaking, thermal cycling, accelerated aging, or Pb leaching tests were performed; the encapsulation was applied as a standard protective measure during characterization and has not been validated as a long-term barrier [46,47]. Systematic stability characterization—including light soaking, thermal cycling (−20 °C to +85 °C), damp-heat testing (85 °C/85% RH), and Pb leaching quantification—is identified as essential future work prior to any practical deployment [48,49,50].

## 3. Results

### 3.1. Optical Characterization of CsPbBr_3_

The optical properties of CsPbBr_3_ perovskite nanoparticles were evaluated using photoluminescence (PL) spectroscopy and UV–Visible (UV-Vis) absorption spectroscopy.

**Figure 3:**
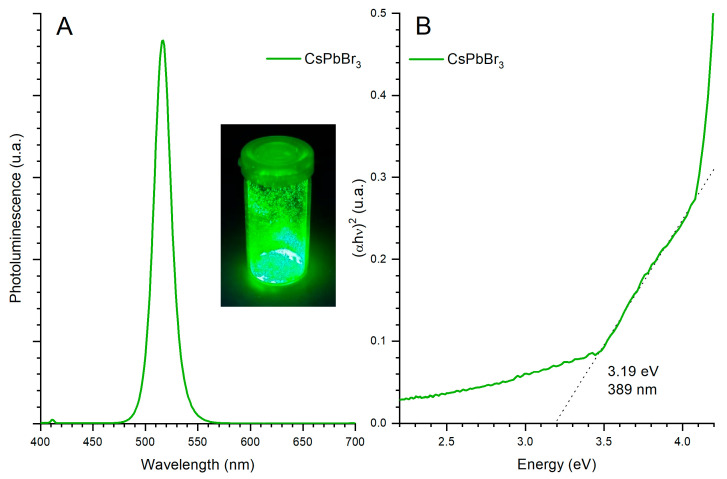
(**A**) Photoluminescence spectrum of the CsPbBr_3_ perovskite nanoparticles excited at 365 nm. The inset shows the colloidal suspension under UV illumination, exhibiting its characteristic green luminescence. (**B**) UV–Vis absorption spectrum of the perovskite nanoparticles.

In Figure 3A, the photoluminescence spectrum of the nanoparticles excited at 365 nm is presented. The spectrum exhibits an intense and symmetric emission centered at 517 nm, which is characteristic of CsPbBr_3_-type perovskites. The emission peak exhibits a FWHM of ~20 nm, consistent with values reported in the literature for CsPbBr_3_ nanocrystals synthesized by antisolvent methods [44], suggesting a relatively homogeneous size distribution. However, definitive assessment of surface defect density requires time-resolved photoluminescence (TRPL) characterization [51]. Figure 3B shows the UV–Vis absorption spectrum of the same nanoparticles. The absorption edge is located at approximately 389 nm, corresponding to a bandgap energy of 3.19 eV, determined from the extrapolation of the Tauc plot for an allowed direct transition, (αhν)^2^ vs. hν. This bandgap value is consistent with those reported in the literature for quantum-sized CsPbBr3 nanoparticles [52].

### 3.2. Characterization of Hybrid Photodetectors

**Figure 4:**
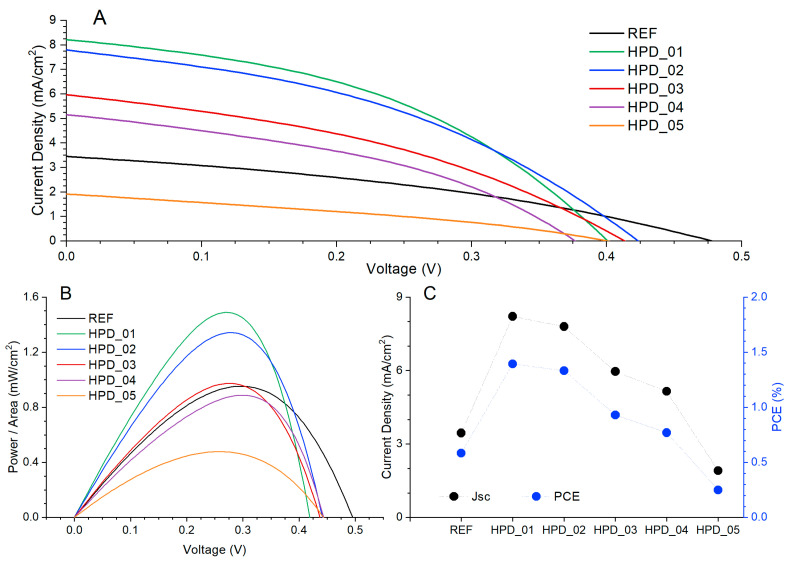
(**A**) J–V curves under illumination (100 mW·cm^−2^) for devices with different PNC concentrations. The active area is 0.06 cm^2^, (**B**) power (P/Area) curves as a function of applied voltage for devices with different concentrations of perovskite nanoparticles and (**C**) variation of the short-circuit current density (black) and power conversion efficiency (blue) as a function of the perovskite nanoparticle concentration in devices.

Figure 4A shows the current density–voltage (J–V) curves measured under illumination for devices with the structure ITO/PEDOT:PSS/P3HT:PCBM: PNCs/Al, incorporating different concentrations of perovskite nanoparticles in the active layer. Measurements were performed using a xenon lamp solar simulator was purchased from Newport Corporation (Irvine, CA, USA) at an irradiance intensity of 100 mW·cm^−2^ (AM1.5G) and an active area of 0.06 cm^2^. From the J–V curves, the characteristic photovoltaic parameters were determined: open-circuit voltage, short-circuit current density, maximum power (P_max_), fill factor (FF), Equation (1), and power conversion efficiency, Equation (2). These values were obtained following conventional relationships:

(1) $$FF=\frac{P_{max}/A}{J_{SC}V_{OC}}$$

(2) $$PCE=\frac{P_{max}}{P_{in}\cdot A}\cdot 100$$

where incident power P_in_ = 100 mW·cm^−2^ and A, area, is 0.06 cm^2^. The parameters extracted from the J–V curves are summarized in Table 2.

As observed, the incorporation of perovskite nanoparticles significantly modifies the photovoltaic response of the devices. The reference device without PNCs exhibits a VOC of 0.478 V and a JSC of 3.451 mA·cm^−2^, reaching a power conversion efficiency of 0.58%. Upon introducing a low concentration of PNCs (HPD_01), a substantial increase in the short-circuit current density (8.21 mA·cm^−2^) and fill factor (42.35%) is observed, leading to an efficiency increase up to 1.39%. This result suggests that moderate addition of nanoparticles enhances the effective optical absorption or facilitates energy transfer effects between the polymer matrix and the PNCs. However, increasing the PNC concentration beyond the optimal point (HPD_03–HPD_05) results in a progressive reduction in JSC and PCE, accompanied by a drop in FF for the device with the highest PNC load (HPD_05). This behavior may be consistent with nanoparticle aggregation within the organic matrix, which could increase series resistance through deterioration of the active layer morphology [53,54], although direct morphological evidence has not been obtained in the present work. These results demonstrate that there is a low optimal PNC concentration that improves the photovoltaic performance of the device without compromising its electrical integrity. Above this concentration, adverse effects associated with nanoparticle dispersion and aggregation dominate, limiting the power conversion efficiency.

Figure 4B shows the dependence of the normalized power (Power/Area) as a function of the applied voltage for the different organic photodetectors. From the J–V curves under illumination, the power was calculated as P = J·V, to obtain P/Area (mW·cm^−2^). The curves exhibit the typical behavior of these devices, with an initial increase in power with applied voltage, reaching a maximum at the maximum power point, and subsequently decreasing. The reference device (REF) reaches a maximum power of 0.584 mW·cm^−2^. This value is used as a benchmark to evaluate the effect of perovskite nanoparticle incorporation into the active layer. With the introduction of PNCs at low concentration (HPD_01 and HPD_02), the maximum power increases significantly, reaching 1.394 and 1.312 mW·cm^−2^, respectively. These values are consistent with the previously calculated efficiencies (1.39% and 1.31%), confirming the agreement between the power curves and the photovoltaic parameters derived from the J–V measurements. This increase is associated with a simultaneous improvement in JSC and FF, resulting from more efficient separation and transport of photogenerated charges, attributable to the effect of the PNCs. As the PNC concentration increases (HPD_03 to HPD_05), a progressive decrease in power/area is observed, reaching a minimum value of 0.249 mW·cm^−2^ in HPD_05. This decline is consistent with the reductions observed in JSC and PCE, and may be tentatively attributed to nanoparticle aggregation and increased defect formation within the P3HT:PCBM matrix, which could reduce charge mobility [55]; however, morphological confirmation is required.

Figure 4C shows the evolution of JSC and PCE as a function of the perovskite nanoparticle concentration incorporated into the active layer of the devices. The reference device, without nanoparticles, exhibits a JSC of 3.451 mA·cm^−2^ and a PCE of 0.58%. Upon incorporation of a low concentration of PNCs (HPD_01 and HPD_02), both JSC and PCE increase significantly, reaching maxima of 8.21 mA·cm^−2^ and 1.39%, respectively. This increase indicates that the moderate addition of perovskite nanoparticles enhances the generation and extraction of photogenerated carriers, possibly through energy or charge transfer mechanisms from the PNCs to the P3HT:PCBM matrix. As the PNC content is further increased (HPD_03 to HPD_05), a progressive decrease in JSC and PCE is observed, reaching minimum values of 1.916 mA·cm^−2^ and 0.25% in HPD_05. This performance degradation is tentatively attributed to aggregation effects and defect formation within the organic matrix caused by the nanocrystals, which may hinder charge transport; AFM and XRD characterization would be required to confirm this hypothesis [55]. At high concentrations, the PNCs cease to act as performance enhancers and begin to function as barriers to carrier flow, reducing the overall device efficiency. These results confirm that the inclusion of perovskite nanoparticles exerts a dual effect on the optoelectronic properties of the system: beneficial at low concentrations, where current generation and efficiency are improved; and detrimental at high concentrations, where the excessive presence of nanoparticles perturbs the morphology and charge transport pathways.

**Figure 5:**
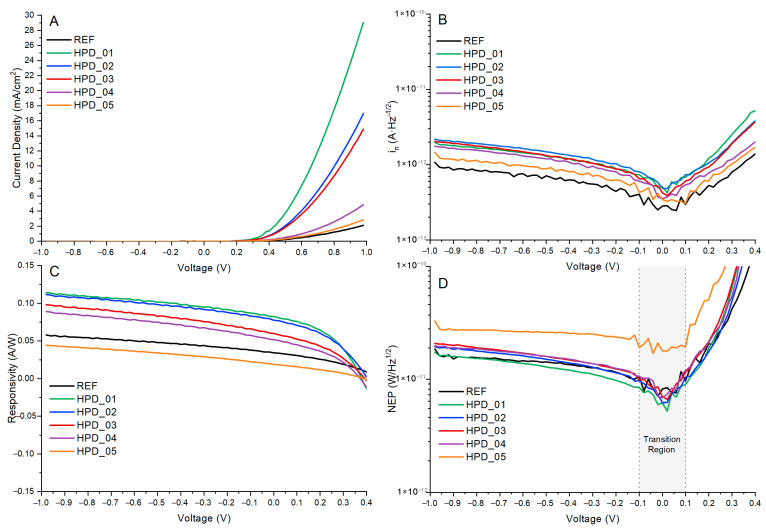
Current–density versus voltage curves under dark conditions (**A**), noise current at 1 Hz as a function of applied voltage (**B**), responsivity as a function of applied voltage (**C**), and NEP dependence (in W·Hz^−1/2^) (**D**) as a function of applied voltage for the fabricated HPD devices.

Figure 5A shows the current–density versus voltage curves measured in dark conditions for the REF and HPD_01–HPD_05 devices. This analysis allows the evaluation of the intrinsic conduction characteristics and leakage current behavior of the photodiodes in the absence of illumination. As observed, all curves exhibit the typical exponential behavior of a diode, with a very low current region at voltages near 0 V and a pronounced increase as the forward bias is raised. The reference device displays the lowest current density across the entire voltage range, showing a controlled response with no significant indications of leakage.

To quantify this behavior, the current densities were compared at V = +0.98 V, where the differences between devices are most pronounced. From the experimental data, the measured currents were approximately 2 mA·cm^−2^ for REF, while the devices modified with perovskite nanoparticles exhibited increases: 29.0 mA·cm^−2^ (HPD_01), 16.9 mA·cm^−2^ (HPD_02), 14.9 mA·cm^−2^ (HPD_03), 4.9 mA·cm^−2^ (HPD_04), and 2.9 mA·cm^−2^ (HPD_05). These values were read directly from the experimental J–V curves, taking the value closest to 1 V for each sample. The results reveal a significant increase in the dark current compared to the reference device, particularly for the HPD_01 and HPD_03 configurations, where the current density is enhanced by a factor of 4 to 5. The increase in dark current could be attributed to an improvement in the conductivity of the P3HT:PCBM active layer following the incorporation of perovskite nanoparticles. In terms of performance, a moderately elevated dark current can be beneficial, as it implies lower series resistance and, consequently, more efficient charge extraction. However, an excessively high dark current could indicate a simultaneous increase in shot noise.

The current noise spectral density (i_n_) represents the statistical fluctuation of the electrical current in the absence of an optical signal and constitutes one of the most relevant parameters for characterizing the electronic quality of a photodetector. In this study, the noise current behavior at a frequency of 1 Hz was analyzed for all devices (REF and HPD_01–HPD_05) under dark conditions and at room temperature. The total noise current spectral density is defined as:

(3) $$i_{n}=\sqrt{i_{shot}^{2}+i_{Johnson}^{2}}$$

where

(4) $$i_{shot}=\sqrt{2q|I_{dark}|\Delta f}$$

where q is the electron charge (1.602 × 10^−19^ C), Id is the dark current, and Δf is the bandwidth (1 Hz in this case). And

(5) $$i_{Johnson}=\sqrt{\frac{4k_{B}T}{R_{Shunt}}}$$

with k_B_ the Boltzmann constant, T = 300 K, and R_shunt_ extracted individually for each device. Equations (4) and (5) are both evaluated as single-sided spectral densities at Δf = 1 Hz, ensuring that all noise quantities carry units of A·Hz^−1/2^ throughout and are not mixed with bandwidth-integrated RMS values.

Figure 5B shows the evolution of the noise current at 1 Hz as a function of the applied voltage for the six analyzed devices. In all cases, the noise progressively increases under forward bias, reflecting the growth of the conduction current. However, the negative-voltage region displays a clearly differentiated behavior that is relevant for applications operating under reverse bias or near-zero applied voltage. In this region (V < 0), the noise remains substantially lower and relatively flat, with a slight downward trend toward a minimum around V ≈ 0 to −0.02 V, indicating reduced leakage currents and a dominance of 1/f or thermal noise rather than the shot noise that prevails under forward bias. Here, the REF device exhibits the lowest average noise levels, followed closely by HPD_05, whereas HPD_02 shows the highest values under negative bias. At low positive voltages (V < 0.1 V), the in values remain on the order of 10^−13^ A·Hz^−1/2^ for all devices, confirming stable electronic behavior and minimal leakage. As the voltage increases toward forward bias, clear differences among the configurations become evident. The REF device exhibits a regular and controlled evolution of the noise, serving as a baseline reference for a device without nanoparticles. HPD_01 presents the highest noise density across the entire positive-voltage range, with a pronounced rise at high bias, which correlates with the larger dark current observed in the J–V curves. HPD_02 and HPD_03 display intermediate responses—HPD_02 slightly more balanced and HPD_03 with a moderate slope—suggesting a relative reduction in surface traps and more controlled charge transport. HPD_04 exhibits lower noise levels at high forward voltages, and HPD_05 shows the lowest values in the high-voltage region, indicating improvements in the suppression of electronic noise. A clear correlation emerges between noise level and dark current: devices with higher conduction (mainly HPD_01 and HPD_03) exhibit higher in values, indicating the predominance of shot noise, whereas those with lower dark current (REF, HPD_04, and HPD_05) show a behavior more strongly influenced by 1/f noise, associated with charge traps in the active layer. Therefore, the selection of the optimal photodetector depends on the operating regime: for reverse-bias or near-zero operation, REF (and secondarily HPD_05) demonstrates superior performance due to its lower noise levels under negative bias. This analysis confirms that optimizing the nanoparticle concentration allows effective control over defect density and, consequently, reduction in electronic noise—an essential factor for improving the D* and the signal-to-noise ratio in organic photodetectors.

Responsivity is a key parameter for evaluating the photoelectric conversion efficiency of a photodetector, as it establishes the relationship between the photocurrent and the incident optical power. Mathematically, it is defined as (Equation (6)):

(6) $$R=\frac{I_{ph}}{P_{inc}\cdot A}$$

where I_ph_ is the photogenerated current, P_inc_ is the incident power per unit area (W·cm^−2^), and A the active area of the device (in this case, 0.06 cm^2^). This parameter, expressed in A·W^−1^, quantifies the ability of the device to convert incident radiation into an electrical signal, and its dependence on the applied bias reflects the internal processes of charge separation and transport within the active layer.

Figure 5C presents the evolution of the responsivity as a function of the applied voltage for the fabricated photodetectors. The curves display the characteristic behavior of P3HT:PCBM-based organic photodetectors, where the responsivity gradually increases under forward bias due to reduced recombination losses and improved extraction of photogenerated carriers. In contrast, under reverse bias the variations are significantly smoother, reflecting a transport regime limited by extraction barriers and lower carrier mobility.

Near 0 V, the responsivity remains relatively low, ranging from approximately 0.019 A·W^−1^ for HPD_05 to 0.034 A·W^−1^ for the reference device. As the voltage increases toward positive values, R exhibits an almost linear rise up to around 0.4–0.5 V, indicating a region in which the photogenerated current is proportional to the incident optical power and where photogeneration clearly dominates over recombination. Above 0.5 V, differences among the devices become increasingly pronounced. Under forward bias above 0.5 V, the net photocurrent becomes negative for all devices as the dark injection current exceeds the photogenerated current, placing the device outside the photodetection regime. Consequently, the practically relevant responsivity values are those in the reverse-bias and self-powered regions. In the reverse-bias region (V = −0.98 V), HPD_01 achieves the highest responsivity of 0.114 A·W^−1^, compared to 0.058 A·W^−1^ for the reference device. HPD_02 shows comparable performance at 0.112 A·W^−1^, while HPD_03, HPD_04, and HPD_05 reach 0.098 A·W^−1^, 0.089 A·W^−1^, and 0.045 A·W^−1^, respectively. This dispersion among the devices suggests that both excessively high and insufficient nanoparticle concentrations may disrupt the balance between carrier transport and recombination, thereby reducing charge-collection efficiency. In the negative-bias region, the responsivity exhibits a much more gradual evolution. For voltages between −1.0 V and −0.4 V, R remains nearly constant or slightly decreases, typically lying between 0.04 A·W^−1^ and 0.11 A·W^−1^ depending on the device. This behavior is consistent with a regime in which the extraction of photogenerated carriers is hindered by an increased effective barrier. Differences between devices are therefore much less pronounced than under forward bias. Nevertheless, HPD_01 and HPD_03 maintain slightly higher responsivities in this region, suggesting that certain doping levels can mildly enhance transport even under reverse bias, although with a far weaker impact compared to forward operation.

A numerical evaluation of the curves confirms that the increase in R under forward bias follows a moderate exponential trend, arising from the combined effects of increased photogenerated current and a reduction in the device’s effective series resistance. This trend aligns with the illuminated J–V characteristics, where devices exhibiting higher photocurrents (notably HPD_01 and HPD_02) also display the highest responsivities. Overall, these results demonstrate that the controlled incorporation of nanoparticles into the active layer significantly enhances optical sensitivity by simultaneously optimizing absorption, transport, and charge-collection processes. The responsivity values achieved by HPD_01 and HPD_02 fall within the typical range of high-performance hybrid organic photodetectors, confirming the effectiveness of the implemented doping strategy.

The NEP parameter is used to quantify the minimum detectable optical signal of a photodetector, expressing the lowest optical power that the device can detect with a signal-to-noise ratio of one within a 1 Hz bandwidth. In general terms, the NEP is obtained from the ratio between the noise current spectral density in and the spectral responsivity R, according to Equation (7)

(7) $$NEP=\frac{i_{n}}{R}$$

The reduction in the NEP value is directly associated with an increase in photodetector sensitivity, as the device becomes capable of distinguishing weaker optical signals above its intrinsic noise background. In organic photodetectors, this parameter acquires particular relevance because it reflects the combined contribution of electronic noise, quantum efficiency, and the charge-transport properties of the active materials. When nanoparticles are incorporated into the device architecture, the NEP additionally becomes a critical indicator of the balance between the enhancement of optical absorption and the potential rise in noise associated with scattering phenomena or carrier-trapping processes. Both metallic and dielectric nanoparticles can simultaneously modify the optical response and the noise characteristics of the photodetector, making NEP analysis essential for evaluating the net impact of such structural modifications. It is worth noting that the NEP values reported in the original version of this manuscript were physically implausible, as they resulted from combining iₙ and R evaluated at different operating voltages. This inconsistency has been corrected in the present version, as detailed in the description of the D* calculation below.

The experimental evolution of NEP as a function of the applied voltage, Figure 5D, for both the reference device and the nanoparticle-modified photodetectors (REF to HPD_05), reveals distinct behaviors depending on the polarization regime. Under negative bias, all devices exhibit moderate and relatively stable values, typically between 5 × 10^−12^ and 2 × 10^−11^ W·Hz^−1/2^, indicating an operating regime in which electronic noise remains well controlled and no marked instability region appears. In this range, the differences among the various HPDs are modest, and no abrupt peaks are observed, suggesting that the incorporation of nanoparticles does not significantly deteriorate performance under reverse bias. This uniform behavior at negative voltages demonstrates that noise-generation mechanisms are mainly limited by intrinsic transport processes rather than by additional effects introduced by the nanoparticles.

In contrast, under positive bias, the devices show a much more pronounced variation. As the voltage approaches the 0.3–0.4 V region, all photodetectors exhibit a characteristic NEP peak, associated with an instability zone in which electronic noise reaches its maximum value. This phenomenon may be attributed to critical transitions in the photocurrent–noise relationship, possibly linked to charge-injection processes, the densification of intermediate states, or the activation of additional recombination channels. Among all devices, HPD_01 displays the highest NEP in the forward bias region, consistent with its higher dark current, substantially exceeding those of the remaining devices, indicating a strong increase in noise likely associated with a higher density of traps or instabilities induced by the specific nanoparticles used in that device.

However, once this critical region is surpassed and voltages higher than 0.5 V are applied, NEP values stabilize, defining a robust photodetection regime in which the photogenerated current clearly dominates over noise-related processes. In this stable interval, the devices exhibit values between 5 × 10^−12^ and 2 × 10^−11^ W·Hz^−1/2^, with HPD_01 showing the minimum NEP of ~5.2 × 10^−12^ W·Hz^−1/2^, consistent with its optimal PNC concentration. This trend indicates that, despite the noise increases observed in the unstable region, the incorporation of nanoparticles can, in some cases, lead to a favorable compromise between enhanced optical absorption and effective noise control. Among all devices, HPD_01 achieves the minimum NEP of ~5.2 × 10^−12^ W·Hz^−1/2^ at the self-powered operating point (V ≈ +0.02 V), confirming that the optimal PNC concentration produces the most favorable balance between enhanced photocurrent and noise floor. Devices with higher PNC loadings (HPD_03–HPD_05) exhibit progressively higher NEP values, consistent with increased noise associated with nanoparticle aggregation and trap-state formation. Overall, the results demonstrate that although the presence of nanoparticles can increase noise in certain voltage regions, their appropriate design enables the development of photodetectors with more controlled NEP behavior and enhanced sensitivity under practical operating conditions.

The specific detectivity Is also a key parameter for evaluating a photodetector’s ability to discriminate weak optical signals against the intrinsic electrical noise of the device. This parameter combines the responsivity with the noise current level and is defined as (Equation (8)):

(8) $$D^{*}=\frac{R\sqrt{A\cdot \Delta f}}{i_{n}}$$

where R is the responsivity in A·W^−1^, A is the active area of the device, Δf is the measurement bandwidth (1 Hz), and i_n_ is the total noise current (A·Hz^−1/2^). The unit used is Jones (cm·Hz^−1/2^·W^−1^), which allows normalized comparison of devices with different sizes and bandwidths. In Equation (8), Δf = 1 Hz is set to unity following the standard convention for specific detectivity, so that D* is expressed in Jones units (cm·Hz^1^/^2^·W^−1^) and represents a bandwidth-normalized figure of merit. The quantity i_n_ is a spectral noise density in A·Hz^−1/2^, not a bandwidth-integrated RMS current, ensuring dimensional consistency throughout.

Since the shunt resistance of the fabricated devices, extracted from the slope of the dark J–V curves near 0 V, ranges from approximately 87 kΩ (HPD_02) to 372 kΩ (REF), the Johnson–Nyquist noise contribution is non-negligible near the self-powered operating point. Therefore, the total noise current was estimated as Equation (3), where i_shot_ is defined in Equation (4), and i_Johnson_ is the Johnson–Nyquist noise current, explained in Equation (5). This analytical approach includes the Johnson–Nyquist contribution explicitly, which is non-negligible given the shunt resistances of our devices (87–372 kΩ), and is therefore more rigorous than the shot-noise-only approximation identified as a source of D* overestimation in the literature [56]. All figures of merit (D*, NEP, R) are evaluated at the same operating voltage to ensure internal consistency.

**Figure 6:**
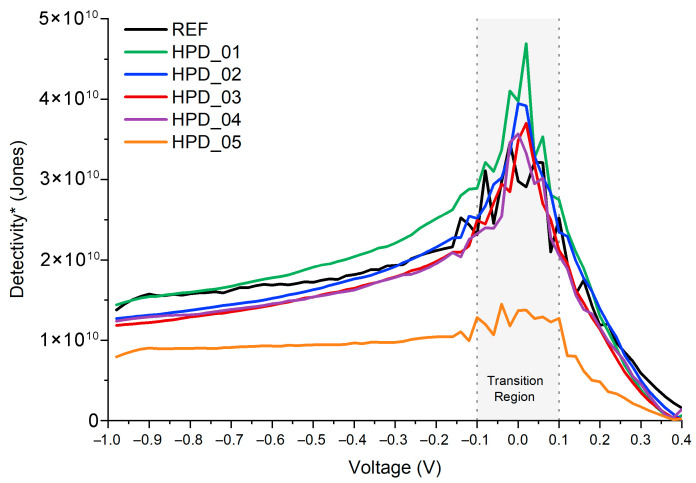
D* of the fabricated devices as a function of applied voltage. The grey shaded region near V = 0 V indicates a mathematical transition zone where the net photocurrent approaches zero and D* diverges; this region does not correspond to a physically meaningful operating point. The operationally relevant D* maximum occurs at the self-powered point (V ≈ 0 V) outside this transition zone.

Figure 6 shows the evolution of D* as a function of the applied voltage for all the evaluated devices.

The evolution of detectivity D* as a function of applied voltage reveals clear differences between the reference device and those modified through the incorporation of nanoparticles, particularly when considering both negative and positive bias regions. Under negative voltages, all photodetectors exhibit moderate and relatively stable values, with D* ranging approximately from 1 × 10^10^ to 2 × 10^10^ Jones, depending on the device. This uniform behavior indicates that, under reverse bias, noise generation mechanisms remain dominated by intrinsic transport processes, and the presence of nanoparticles does not introduce significant additional fluctuations. In this region, the spread among HPD devices is relatively small, suggesting that sensitivity to weak optical signals remains comparable and that no device exhibits electronic instability under negative bias.

As the voltage approaches 0 V, a sharp peak in D* is observed for all devices, particularly pronounced for HPD_01. This feature arises from a mathematical divergence as the net photocurrent approaches zero and responsivity crosses zero, rather than representing a physically meaningful operating point; this region is indicated as a transition zone in Figure 6. The operationally relevant D* maximum for HPD_01, evaluated at the self-powered point (V ≈ +0.02 V), is 4.69 × 10^10^ Jones, reflecting a regime where the combination of enhanced responsivity and moderate noise leads to improved detectivity. The remaining devices also show D* maxima in this voltage interval, with values of 3.95 × 10^10^ (HPD_02), 3.70 × 10^10^ (HPD_03), 3.57 × 10^10^ (HPD_04), and 1.45 × 10^10^ Jones (HPD_05), all exceeding the reference device value of 3.46 × 10^10^ Jones except HPD_05. This ordering is consistent with enhanced charge-transfer efficiency at low PNC concentrations and progressive degradation at higher loadings.

Under positive bias—particularly from approximately 0.3–0.4 V onward—all devices exhibit a general upward trend in D*, consistent with increased photocurrent generation, stronger internal electric field, and more efficient charge separation. However, the magnitude of this enhancement differs among devices. HPD_01 remains the best-performing device at reverse bias, reaching ~1.4 × 10^10^ Jones at −0.98 V, above the reference device which reaches ~1.3 × 10^10^ Jones at the same voltage. HPD_02 also shows a performance improvement compared to the reference at intermediate voltages, although its D* decreases more significantly approaching 0.98 V. In contrast, devices with higher nanoparticle concentrations (HPD_04 and HPD_05) do not achieve high detectivity at positive bias. While they may exhibit increased responsivity, their D* values at higher voltages remain in the range of 5–15 × 10^9^ Jones, limited by elevated noise levels and increased dark current. This behavior is consistent with nanoparticle aggregation and the formation of additional trap states as a plausible mechanism [55], though direct structural evidence is not available in the present work.

Overall, the D*–V relationship demonstrates a clear trade-off between optical absorption, charge transport, and electronic noise. The results confirm that only controlled nanoparticle incorporation—particularly at low concentrations—enhances detectivity across the full operating range, whereas excessive nanoparticle loading compromises device performance. Among all devices, HPD_01 consistently exhibits the best overall detectivity, followed by HPD_02, while higher nanoparticle concentrations significantly reduce the photodetector’s capability to achieve high sensitivity under practical operating conditions. The SNR as a function of voltage provides a direct measure of the relationship between the useful signal and the noise of the photodetector, which is particularly relevant for applications under practical operating conditions. In this case, the SNR has been calculated as Equation (9), under the following measurement conditions: xenon lamp solar simulator (100 mW·cm^−2^, AM1.5G), active area A = 0.06 cm^2^, noise bandwidth Δf = 1 Hz, and T = 300 K.

(9) $$SNR=\frac{I_{ph}-I_{dark}}{i_{n}}$$

where I_ph_ is the current under illumination, I_dark_ is the dark current measured without illumination, and in corresponds to the noise current density at the applied voltage V. This approach makes it possible to evaluate the efficiency of the device in distinguishing the photogenerated signal from the inherent noise level of the system, considering the contribution of the dark current.

**Figure 7:**
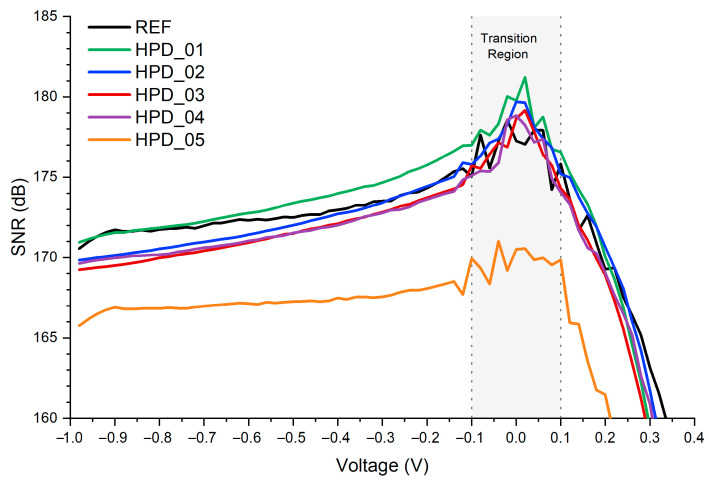
Signal-to-noise ratio expressed in decibels as a function of the applied bias for all fabricated photodetectors.

Analyzing the SNR expressed in decibels (Figure 7), it is observed that all devices exhibit their highest values in the low-bias range, approximately between −0.02 V and 0.08 V, where the signal clearly dominates over the noise. In this region, HPD_01 reaches the maximum SNR, with a value of approximately 181 dB at 0.02 V, outperforming the reference device, which shows a peak of ~179 dB. The other detectors exhibit slightly lower maxima, with values of ~180 dB for HPD_02, ~179 dB for HPD_03, ~179 dB for HPD_04, and ~171 dB for HPD_05, indicating that all devices achieve their optimal noise performance in this low-voltage range.

When studying higher negative voltages, in the range from −0.98 V to −0.1 V, an interesting and distinctive behavior among the devices is observed. In this regime, the SNR is very high, on the order of 107–108 in linear units, reflecting a highly favorable and relatively stable signal-to-noise relationship. HPD_01 and HPD_02 show progressive increases in SNR as the voltage approaches −0.2 V, reaching values higher than the reference, which demonstrates the effectiveness of nanoparticles in enhancing charge generation and transport efficiency without introducing significant noise. In contrast, HPD_04 and HPD_05 exhibit a more moderate increase in SNR, indicating that higher nanoparticle concentrations induce an increase in electronic noise, possibly associated with particle aggregation or the generation of additional trap states, thereby limiting effective sensitivity. HPD_03 behaves intermediately, showing a constant upward trend but without surpassing the SNR values of HPD_01 and HPD_02. As the voltage approaches 0 V from negative bias, HPD_01 reaches a pronounced maximum, evidencing a regime in which low noise density and high responsivity combine to generate an exceptionally high SNR. At this point, HPD_02 also exhibits a very high value, albeit slightly lower, while HPD_03–HPD_05 show more moderate peaks. This behavior highlights the importance of precise control in nanoparticle incorporation: low concentrations allow for SNR maximization without compromising electronic integrity, whereas high concentrations limit performance.

Under positive bias, starting from 0.1 V, the SNR of all detectors progressively decreases, reaching a minimum around 0.4–0.45 V, where the combined effects of dark current and noise become dominant. In this region, the minimum SNR values drop to approximately 143–157 dB depending on the device, reflecting a clear degradation in signal quality. Beyond this point, the SNR gradually recovers up to 1 V, stabilizing in the 64–68 dB range at high voltages. HPD_01 consistently maintains the highest SNR throughout the entire voltage sweep, although the differences relative to HPD_02 and HPD_03 diminish above 0.6 V. From a practical perspective, HPD_01 exhibits the peak SNR, while HPD_02 and HPD_03 demonstrate a more stable and homogeneous behavior across the full voltage range, with moderate decreases and smooth transitions. In contrast, HPD_04 and HPD_05 show lower SNR values and steeper degradation, indicating a stronger influence of noise and limiting their sensitivity to weak optical signals.

### 3.3. Distance–Frequency Voltage in VLC Conditions

To evaluate the performance of the photodetectors with respect to the emission frequency, the following experimental setup was used: the system was configured to determine the maximum frequency supported by a VLC system. The transmitter consisted of a high-brightness white phosphor-based LED with a correlated color temperature of 5500 K. An arbitrary waveform generator (AWG, Digilent^®^ Analog Discovery 2, Pullman, Washington, DC, USA) was used to generate the signals, which were applied to the LED. The AWG operated with an analog bandwidth of 12 MHz and a sampling rate of 100 MSamples/s. The generated VLC signal was transmitted over a fixed distance and detected by the self-powered HPD. This OPD was connected to a real-time oscilloscope (RTO, Digilent^®^ Analog Discovery 2) for signal detection. The RTO featured an analog bandwidth of 30 MHz and a sampling rate of 100 MSamples/s, ensuring accurate signal detection and analysis. From the frequency-dependent voltage data recorded at 0 cm, the −3 dB bandwidth of each device was extracted by normalizing the detected voltage to its value at 1 kHz and identifying the frequency at which the normalized response drops to 0.707, using log-linear interpolation. The resulting −3 dB bandwidths are 8.4 kHz (REF), 17.0 kHz (HPD_01), 11.4 kHz (HPD_02), 10.0 kHz (HPD_03), 10.2 kHz (HPD_04), and 7.9 kHz (HPD_05), demonstrating that HPD_01 achieves approximately twice the bandwidth of the reference device.

**Figure 8:**
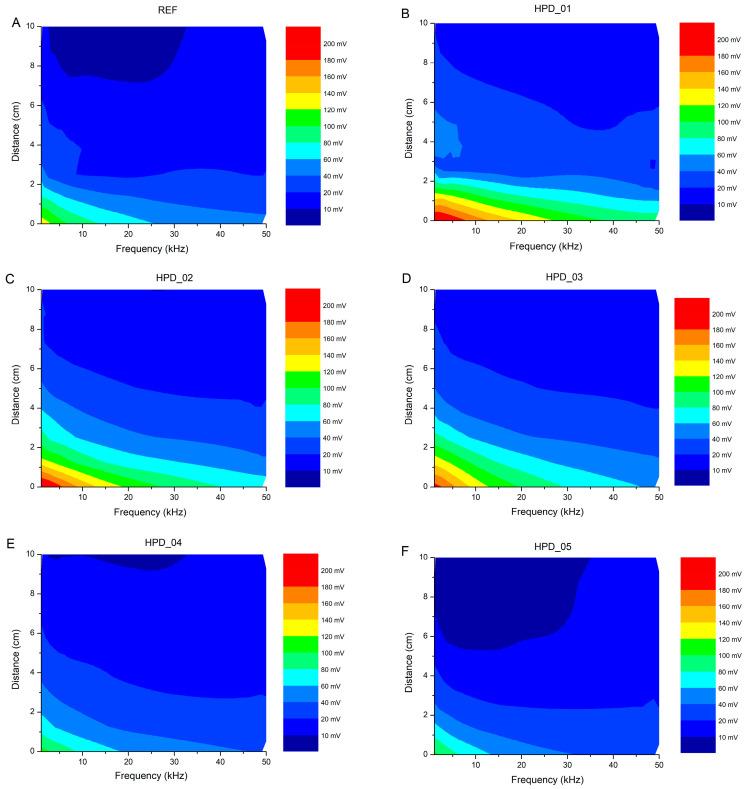
The color map illustrates the variation of the detected signal as a function of the distance and the emitter frequency, highlighting the maximum voltage at low frequencies and short distances, and a progressive decrease as both parameters increase. Voltage heatmap for REF (**A**), for HPD_01 (**B**), for HPD_02 (**C**), for HPD_03 (**D**), for HPD_04 (**E**) and for HPD_05 (**F**).

**Figure 9:**
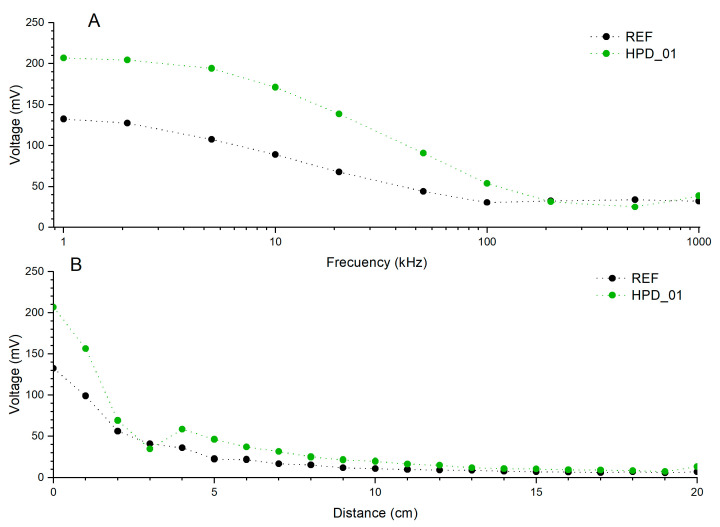
(**A**) Voltage dependence on modulation frequency (kHz) at a distance of 0 cm for the REF (black) and HPD_01 (green) devices. (**B**) Variation of the detected voltage with distance (cm) at a modulation frequency of 1 kHz for the same devices.

The VLC characterization is presented in two complementary parts with distinct objectives. First, Figure 8 presents voltage heatmaps covering 1–50 kHz and 0–10 cm for all six devices, enabling systematic comparison across the full concentration series within the IEEE 802.15.7 VLC band [57]. Second, Figure 9A presents a dedicated frequency sweep from 1 kHz to 1 MHz for HPD_01 and REF only, performed to characterize the high-frequency roll-off and upper detection limit of the optimized device. The −3 dB bandwidth values (8.4 kHz for REF; 17.0 kHz for HPD_01) are the operative VLC figures of merit; the 1 MHz measurement establishes the upper detection limit, not the operational bandwidth. For a first-order RC-limited system, the rise time is related to the −3 dB bandwidth by t_r_ ≈ 0.35/f_−3dB_ [55], giving estimated rise times of ~21 µs for HPD_01 and ~42 µs for REF. The frequency-domain characterization methodology follows standard practice for OPD-based VLC receivers [15]. Direct time-domain measurements under pulsed illumination are identified as future work to provide a more rigorous temporal characterization.

The results obtained for the fabricated devices are presented as a voltage heatmap in Figure 8, where the X-axis indicates the frequency of the LED signal (in kHz), the Y-axis represents the distance between the HPD and the emitter (in cm), and the color reflects the voltage recorded by the photodetector (in mV). This type of representation allows a clear visualization of how the detected signal varies with both distance and excitation frequency.

By analyzing the heatmap (Figure 8A), it is observed that the received voltage clearly decreases as the distance between the HPD and the LED emitter increases. This behavior is consistent with the inverse-square law and with coupling losses in the optical system, as the intensity of light reaching the photodetector decreases with distance, resulting in lower voltages. For example, at 1 kHz, the voltage drops from ~132 mV at 0 cm to ~10–12 mV at 10 cm, showing a pronounced decline. Regarding the emitter frequency, higher voltages are observed at low frequencies (1–2 kHz) and progressively decrease as the frequency increases. This can be attributed to limitations in the HPD and readout circuit frequency response, as well as capacitance effects and the photodiode response times, which attenuate fast signals. For instance, at 50 kHz, even at short distances, the maximum voltage is ~44 mV, significantly lower than the ~132 mV at 1 kHz. The heatmap also suggests some nonlinearity at intermediate distances: for medium frequencies (5–10 kHz), the voltage does not decrease strictly monotonically with distance, which could be due to optical interference, internal reflection, or scattering effects in the OPD encapsulation. At high frequencies and long distances (20–50 kHz, 7–10 cm), the signal approaches the noise level, evidenced by low voltages (~7–15 mV).

The results obtained for HPD_01 are presented as a voltage heatmap in Figure 8B. Analyzing the heatmap, the received voltage generally decreases with distance, though less uniformly than in REF. At 1 kHz, the maximum voltage is at 0 cm (~207 mV) and decreases to ~20 mV at 10 cm, showing a marked drop with distance, consistent with the inverse-square law and optical coupling losses. However, at intermediate distances (3–5 cm), relatively higher voltage peaks are observed (58.55 mV at 4 cm), indicating possible optical interference, internal reflection, or scattering in the HPD encapsulation. Regarding the emitter frequency, HPD_01 maintains high voltages at low frequencies (1–2 kHz), but the response decreases progressively with increasing frequency, reflecting the frequency response limitations of the HPD and readout circuit. For example, at 50 kHz, even at short distances, the maximum voltage barely reaches ~91 mV, considerably lower than the ~207 mV at 1 kHz. At high frequencies and long distances (20–50 kHz, 7–10 cm), the signal approaches the noise level, evidenced by low voltages (~11–15 mV). Compared with REF, HPD_01 shows a higher maximum voltage at short distances, indicating a stronger signal level, but also exhibits less uniform variations with distance, suggesting that device geometry, alignment, or encapsulation affect the spatial distribution of the detected signal.

Analyzing the HPD_02 heatmap (Figure 8C), the maximum voltage is reached at 1 kHz and 0 cm (~209 mV), decreasing progressively with distance to ~19 mV at 10 cm. The behavior is fairly uniform, although small voltage irregularities (~43–58 mV) are observed at intermediate distances (3–5 cm), likely due to optical interference or internal reflections. The frequency response of HPD_02 is consistent, with voltages decreasing as frequency increases, reaching ~71 mV at 50 kHz and 0 cm. For HPD_03 (Figure 8D), the maximum voltage at 1 kHz and 0 cm is ~188 mV, decreasing progressively with distance to ~16 mV at 10 cm. Voltage peaks at intermediate distances (2–4 cm) suggest some nonlinearity in the optical distribution of the device. The decline with frequency is clear, with the maximum voltage at 50 kHz not exceeding ~39 mV. HPD_04 (Figure 8E) exhibits a maximum voltage of ~108 mV at 1 kHz and 0 cm, significantly lower than previous HPDs. The drop with distance is more pronounced and relatively linear, reaching ~11–12 mV at 10 cm. The frequency response shows that at 50 kHz, even at short distances, the maximum voltage is ~27 mV, evidencing a greater limitation at high frequencies. In the case of HPD_05 (Figure 8F), the maximum voltage is ~105 mV at 1 kHz and 0 cm. The decrease with distance is gradual, although at intermediate distances, somewhat elevated voltages (~17–25 mV at 4–6 cm) are detected, possibly due to interference or local optical coupling. The frequency response indicates that voltages decrease with increasing frequency, and at 50 kHz, the maximum voltage is ~34 mV. HPD_01 and HPD_02 reach the highest maximum voltages, while HPD_04 and HPD_05 show lower responses, indicating differences in detection efficiency and optical coupling. Additionally, HPDs with higher maximum voltages also present irregularities at intermediate distances, which could indicate scattering or internal reflection effects.

It is observed that the HPDs incorporating nanoparticles, particularly HPD_01 and HPD_02, exhibit significantly higher maximum voltages than REF at low frequencies and short distances, indicating an improvement in light absorption and signal coupling to the photodetector. HPD_03 shows intermediate performance, while HPD_04 and HPD_05 display lower voltages, suggesting that nanoparticle integration in these cases does not optimize light capture, possibly due to particle scattering or aggregation that limits efficiency. In all HPDs, the measured voltage decreases as the frequency increases, reflecting the limited temporal response of the HPD and the readout circuit. Similarly, the voltage decreases with increasing distance between the HPD and the LED emitter, approximately following the inverse-square law, although with some irregularities at intermediate distances that could be attributed to optical interference or internal reflections in the encapsulation. In general terms, the maximum signal is always detected at 1 kHz and 0 cm, Table 3. HPD_01 and HPD_02 stand out for maintaining the best signal-to-distance relationship across the entire measurement range, demonstrating that well-integrated nanoparticles optimize light capture. HPD_03 shows slightly lower performance, while HPD_04 and HPD_05 exhibit more limited behavior, indicating that the mere presence of nanoparticles does not guarantee superior performance if factors such as concentration, size, or dispersion of the particles are not controlled. These results complement the previous analyses of SNR and spectral efficiency of the HPDs, directly linking to the characterization of detector behavior under different experimental conditions and reinforcing the relevance of nanoparticle incorporation for enhancing optical response in organic OPD devices.

### 3.4. Integrated Electrical–Optical Performance Summary

To integrate and globally compare the electrical, optical, and performance parameters obtained for the different fabricated organic photodetectors, a summary table (Table 4) was prepared compiling the most representative values. This combined comparison provides a comprehensive view of the effect of perovskite nanoparticle concentration on the optoelectronic properties and detection capabilities of the hybrid devices.

The comparative analysis of the parameters compiled in Table 4 and Table 5 highlights the direct effect of incorporating CsPbBr_3_ PNCs on the electrical and optical properties of the devices. The results show that the HPD_01 and HPD_02 photodetectors exhibit significantly superior performance compared to the reference device, achieving power conversion efficiencies of 1.39% and 1.31%, respectively, versus 0.58% for the nanoparticle-free device. This increase is accompanied by a notable rise in J_SC_, responsivity (R = 0.083 A·W^−1^ at V ≈ 0 V; 0.114 A·W^−1^ at V = −0.98 V for HPD_01), and specific detectivity (D* ≈ 4.69 × 10^10^ Jones), evidencing an enhanced capability for photogenerated carrier generation and collection. Analysis of the electronic noise confirms that devices doped with low PNC concentrations maintain moderate current spectral noise densities (i_n_ ≈ 5–7 × 10^−13^ A·Hz^−1/2^) and a minimum NEP of 5.2 × 10^−12^ W·Hz^−1/2^ for HPD_01 at the self-powered operating point, implying an optimal balance between signal amplification and electronic stability. Consequently, the signal-to-noise ratio increases substantially, reaching maximum values of ~181 dB for HPD_01 and ~180 dB for HPD_02, compared to ~179 dB for REF. In contrast, at higher nanoparticle concentrations (HPD_03–HPD_05), efficiency, responsivity, and SNR gradually decrease, accompanied by increased noise and internal scattering, confirming the existence of an optimal low-doping point. The photonic behavior shows a clear correlation between electrical performance and the voltage detected under different experimental conditions. At low frequencies and short distances (1 kHz–0 cm), HPD_01 and HPD_02 reach maximum voltages of 206–209 mV, higher than the 132 mV of REF, reflecting more efficient light absorption and energy transfer. At higher frequencies and longer distances (50 kHz–10 cm), voltages decrease for all devices, although HPD_01 and HPD_02 maintain higher values (~11–12 mV), indicating a better temporal response to modulated signals. In contrast, devices with high nanoparticle concentrations (HPD_04 and HPD_05) show lower voltages and greater frequency dependence, associated with recombination and scattering processes.

These results confirm that HPD_01 provides the best overall performance, significantly improving upon the reference device, by combining high photoelectric efficiency with enhanced detectivity, controlled maximum NEP, and stable response over a wide frequency range. This balance between optical gain and noise control demonstrates that the controlled incorporation of perovskite nanoparticles at moderate concentrations is an effective strategy to optimize both the sensitivity and functional stability of hybrid organic photodetectors.

### 3.5. In-Depth Comparative Analysis Between HPD_01 and REF

Given that the HPD_01 device exhibited the best overall performance among all photodetectors evaluated in this work, a more detailed comparative analysis was carried out against the reference device to assess its behavior at longer detection distances and over a broader frequency range. Figure 9 presents two illustrative plots.

The analysis of Figure 9A reveals the typical attenuation of the detected signal with increasing modulation frequency for both photodetectors. At low frequencies (1–10 kHz), both REF and HPD_01 exhibit relatively high voltages; however, the doped device consistently maintains significantly higher values throughout the entire frequency range. At 1 kHz, HPD_01 reaches 206.8 mV compared to 132.5 mV for REF, representing an increase of approximately 56% in the detected signal amplitude. As the modulation frequency increases, the voltage decreases almost exponentially, reaching 90.7 mV (HPD_01) and 44.2 mV (REF) at 50 kHz, and further dropping to 53.8 mV and 30.5 mV, respectively, at 100 kHz. This reduction reflects the intrinsic response-time limitations of organic photodetectors. Nevertheless, HPD_01 maintains a more stable and quasi-linear trend up to 100 kHz, indicating a substantial improvement in dynamic detection performance associated with the incorporation of perovskite nanoparticles, which facilitate charge-transfer processes. At very high frequencies (200–1000 kHz), both devices exhibit a pronounced decay, although with distinct behaviors. While REF shows an almost constant response around 30–33 mV, HPD_01 displays a less uniform decrease, attaining a minimum voltage of 25.0 mV at 500 kHz followed by a slight increase to 38.7 mV at 1 MHz. Overall, these results indicate that HPD_01 maintains voltages approximately twice as high as REF across most of the tested spectrum, confirming its superior ability to track modulated signals. It is noted that the 1 MHz measurement establishes the upper detection limit of the device; the operational VLC bandwidth, defined by the −3 dB criterion, is 17.0 kHz for HPD_01. It is acknowledged that the noise analysis presented in Section 3.2 was performed at 1 Hz, where 1/f noise dominates, and therefore does not directly predict the noise floor in the VLC operating band. A complete characterization of the noise spectral density S(f) across the kHz–MHz range, which would enable the calculation of band-integrated D and NEP values representative of actual VLC receiver performance. It is worth noting that the optical feasibility of the proposed radiative reabsorption pathway would depend on several device-optical factors that have not been quantitatively characterized in the present work. The P3HT:PCBM blend exhibits strong absorption across the 450–650 nm range [58], and the characteristic reabsorption length at 517 nm might be expected to be comparable to the active layer thickness (~100 nm), which could in principle favor reabsorption of re-emitted photons before they escape the device. At the low PNC loading used in HPD_01 (0.42 mg CsPbBr_3_), absorption would be expected to dominate over Mie scattering given the small nanocrystal size relative to the optical wavelength [59], consistent with the performance degradation observed at higher PNC concentrations where aggregation is more likely. Additionally, internal reflection at the Al back contact could contribute to increasing the effective optical path length within the active layer [60], analogously to effects reported in structured P3HT:PCBM films [61]. The concept of embedded perovskite nanocrystals acting as luminescent downshifters within photovoltaic active layers has been discussed in the broader literature [62]. However, all of the above arguments remain qualitative, and a rigorous optical treatment—including transfer-matrix modeling and direct measurement of the blend absorption coefficient at 517 nm would be required to fully confirm this mechanism.

Figure 9B, which represents the variation of voltage with distance at a modulation frequency of 1 kHz, shows a gradual decrease in the detected signal for both devices as the separation between the LED emitter and the photodetector increases, approximately following an inverse-square relationship consistent with free-space light propagation. However, substantial differences are observed between the two devices: while REF decreases from 132.5 mV to 10.9 mV when the distance increases from 0 to 10 cm, HPD_01 maintains higher values across the entire range, with a voltage of 19.9 mV at 10 cm. Additionally, HPD_01 exhibits localized irregularities between 3 and 6 cm, where intermediate voltage peaks appear (58.6 mV at 4 cm and 46.2 mV at 5 cm), possibly attributable to internal reflections or optical interference within the encapsulation—an effect that does not manifest as strongly in the reference device. At larger distances (≥15 cm), HPD_01 still maintains detectable voltages (~10 mV), whereas REF falls below that threshold beyond 12 cm, indicating a higher sensitivity under low irradiance and better utilization of the incident radiation in the modified device. This comparison confirms that HPD_01 exhibits superior performance across the entire range of tested frequencies and distances, maintaining stronger signals, more stable responses, and a more linear behavior under optical modulation. These enhancements are attributed to the controlled incorporation of CsPbBr_3_ perovskite nanoparticles within the active layer, which improve optical absorption, charge mobility, and carrier-extraction efficiency. Such behavior reinforces the potential of HPD_01 as a high-sensitivity and fast-response photodetector, markedly surpassing the dynamic limitations of conventional organic systems.

**Figure 10:**
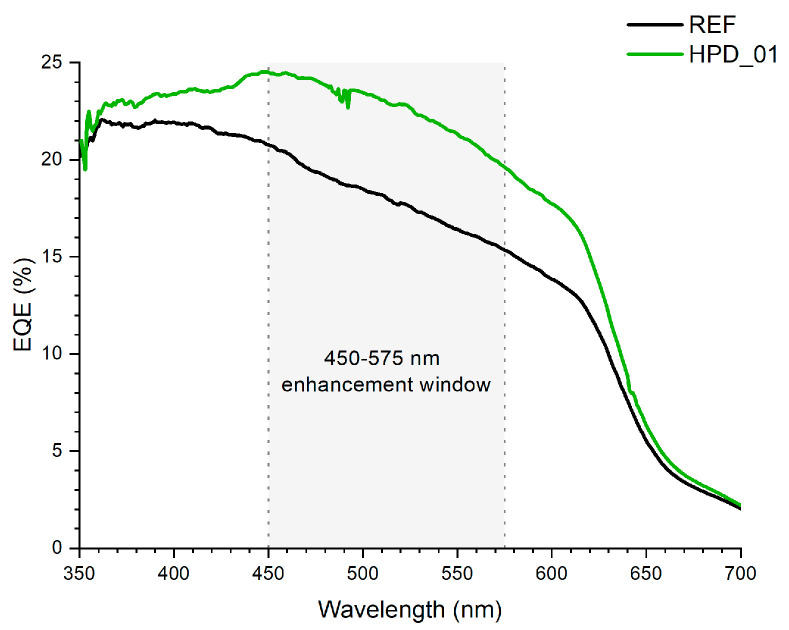
EQE spectra of the REF (black) and HPD_01 (green) devices.

Figure 10 shows the external quantum efficiency spectra of the REF and HPD_01 devices in the 350–700 nm wavelength range. In both cases, an initial maximum is observed around 360–380 nm, followed by a progressive decrease toward longer wavelengths, which is characteristic of photodetectors based on the P3HT:PCBM architecture. The response of the reference device exhibits the typical shape of this system, with an almost monotonic decrease in EQE as the wavelength increases. In contrast, the HPD_01 device exhibits a consistently higher EQE across the entire spectral range. The enhancement is particularly pronounced in the visible region, where the presence of perovskite nanoparticles induces a clear modification in the spectral response. Specifically, between approximately 450 and 575 nm, the HPD_01 curve displays an additional contribution that is not observed in the reference device. This “spectral hump” appears as an elevated plateau in the EQE of the doped device, which remains above the corresponding REF values throughout this interval.

**Figure 11:**
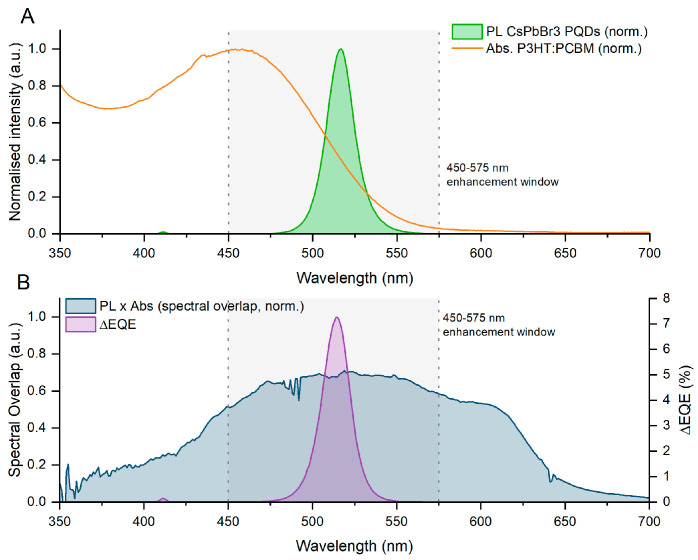
(**A**) shows the normalized PL emission of the CsPbBr_3_ PNCs (green filled area) and the normalized UV–Vis absorption spectrum of the P3HT:PCBM matrix (orange line). (**B**) shows the normalized spectral overlap function PL × Abs and the differential EQE spectrum ΔEQE = EQE_HPD_01–EQE_REF.

This behavior could be directly attributed to the incorporation of CsPbBr_3_ perovskite nanoparticles, whose photoluminescence emission is centered around 517 nm (as shown in the PL spectra included in Figure 3). These PNCs exhibit strong absorption in the ultraviolet and blue regions of the spectrum, followed by an efficient emission process in the green range. To investigate the underlying physical mechanism, a quantitative spectral overlap analysis was performed combining the PL emission spectrum of the CsPbBr_3_ PNCs, the UV–Vis absorption spectrum of the P3HT:PCBM matrix, and the differential EQE spectrum (ΔEQE = EQE_HPD_01 − EQE_REF), as shown in Figure 11. The PL emission of the PNCs is centered at 517 nm with a narrow FWHM of ~20 nm, and 99.5% of this emission falls within the 450–575 nm window where the EQE enhancement is observed. Critically, the maximum ΔEQE occurs at 519 nm, only 2 nm from the PNC emission peak, and the integrated spectral overlap between the PNC emission and the P3HT:PCBM absorption accounts for 26.6% of the total PL emission. The mean EQE enhancement in the 450–575 nm range is +26.1%, with a peak gain of +5.17% at 519 nm. As shown in Figure 11B, the spectral overlap function PL × Abs peaks at 514 nm, and the maximum ΔEQE occurs at 519 nm. The near-coincidence of the PL emission peak (517 nm), the overlap maximum (514 nm) and the ΔEQE maximum (519 nm) within a spectral window of less than 6 nm is strong circumstantial evidence for a radiative reabsorption pathway.

These spectral correlations are consistent with a radiative absorption–reemission pathway, in which PNCs absorb high-energy photons, generate excitons, and re-emit photons around 517 nm that are subsequently reabsorbed by the P3HT:PCBM matrix, introducing an additional charge-generation pathway not present in the reference device. However, EQE and steady-state PL analysis alone cannot unambiguously distinguish this mechanism from non-radiative Förster Resonance Energy Transfer, direct charge transfer at the PNC/organic interface, or morphological changes in the active layer. Time-resolved photoluminescence (TRPL) measurements represent the standard approach to discriminate between radiative and non-radiative energy transfer pathways in quantum dot/organic systems [63], and are identified as essential future work to definitively establish the operative mechanism in this system.

### 3.6. Comparative Analysis with Literature

Table 6 summarizes the key optoelectronic and VLC figures of merit of the devices reported in this work alongside the most relevant P3HT:PCBM-based OPDs and PQD-enhanced hybrid photodetectors reported in the literature.

The most relevant comparison is with Arredondo et al. 2013 [64] and Salamandra et al. 2020 [15], which represent the highest-bandwidth P3HT:PCBM OPDs reported for VLC applications. Both achieve bandwidths in the range of 790 kHz–1 MHz, substantially higher than our devices; however, both require reverse bias operation, which increases power consumption and circuit complexity. Our HPD_01 achieves a −3 dB bandwidth of 17.0 kHz and maintains detectable signals up to 1 MHz at V = 0 V (self-powered), which is competitive within the IEEE 802.15.7 VLC standard operating band [57]. The lower bandwidth relative to biased devices reflects the known RC time constant limitation of self-powered BHJ OPDs and is acknowledged as a trade-off of the self-powered operating regime.

The work of Kim et al. [65] is the most structurally analogous to ours, demonstrating that CsPbBr_3_ NCs can enhance the performance of P3HT-based OPDs. However, that work employs a photomultiplication-type architecture (PM-OPD) under reverse bias, achieving very high EQE and D* but at the cost of bandwidth (~0.5 kHz) due to the fundamental gain–bandwidth trade-off inherent to PM-OPDs. Our approach uses standard photodiode-mode operation at V = 0 V, demonstrating for the first time that low-concentration CsPbBr_3_ NC incorporation in a direct P3HT:PCBM architecture improves both responsivity (+141% vs. REF) and bandwidth (+102% vs. REF) simultaneously under self-powered VLC conditions.

## 4. Conclusions

The incorporation of CsPbBr_3_ PNCs at low concentrations into P3HT:PCBM devices significantly enhances the optoelectronic performance of the organic photodetectors. The optimized HPD_01 device, with controlled nanoparticle loading, achieves a specific detectivity of approximately 4.7 × 10^10^ Jones, a responsivity of 0.083 A/W at the self-powered point (V ≈ 0 V) and 0.114 A·W^−1^ at V = −0.98 V, and a maximum signal-to-noise ratio of ~181 dB, substantially surpassing the reference device without PNCs. Analysis of the EQE spectra reveals an additional contribution in the 450–575 nm range, absent in the reference device. Quantitative spectral overlap analysis (Figure 11) shows that 99.5% of the PNC photoluminescence falls within this enhancement window and that the maximum differential EQE gain occurs at 519 nm, only 2 nm from the PNC emission peak, providing strong circumstantial evidence for a radiative absorption–reemission energy-transfer pathway.

Furthermore, HPD_01 achieves a −3 dB bandwidth of 17.0 kHz, approximately twice that of the reference device, and maintains higher detected voltages across a wide range of modulation frequencies and distances. It is noted that noise characterization in the VLC operating band (kHz–MHz) represents an important direction for future work, as it would enable the calculation of band-integrated figures of merit directly representative of receiver performance. In contrast, devices with higher PNC concentrations show reduced performance tentatively attributed to nanoparticle aggregation, trap formation, and increased noise [53,54], pending morphological characterization, highlighting the critical importance of precise control in nanoparticle incorporation. These results strongly suggest that the controlled integration of PNCs in organic photodetectors not only enhances light harvesting but also promotes efficient photonic energy coupling, yielding devices with high sensitivity and fast response. These results constitute a proof-of-concept demonstration of the potential of PNC-enhanced OPDs for visible light communication applications.

From an environmental perspective, the optimized device HPD_01 contains 150 μg of Pb (0.058 wt% of total device mass), below the RoHS Directive threshold of 0.1 wt% [66]; higher-concentration devices (HPD_02–HPD_05) exceed this limit and would require reformulation for commercial deployment. No Pb leaching or accelerated aging tests were performed in the present work; systematic moisture stability and Pb release characterization are identified as essential steps prior to any practical deployment [49,53].

## Figures and Tables

**Table 1:** Structure and material ratios used in the fabrication of HPDs with different concentrations of perovskite nanoparticles.

Devices	Ratio P3HT:PCBM:PNCs	CsPbBr_3_	PNC Content (wt%)
REF	1:0.8:0.0	0.00 mg	0.00
HPD_01	1:0.8:0.5	0.42 mg	2.06
HPD_02	1:0.8:1.0	0.83 mg	3.98
HPD_03	1:0.8:2.0	1.67 mg	7.71
HPD_04	1:0.8:5.0	4.17 mg	17.25
HPD_05	1:0.8:10.0	8.33 mg	29.40

**Table 2:** Electrical Parameters of the Fabricated Devices.

Devices	V_OC_	J_SC_	P_max_	FF	PCE
REF	0.478 V	3.451 mA·cm^−2^	0.035 mW	35.41%	0.58%
HPD_01	0.401 V	8.210 mA·cm^−2^	0.084 mW	42.35%	1.39%
HPD_02	0.424 V	7.796 mA·cm^−2^	0.079 mW	39.75%	1.31%
HPD_03	0.413 V	5.963 mA·cm^−2^	0.056 mW	37.73%	0.93%
HPD_04	0.377 V	5.149 mA·cm^−2^	0.046 mW	39.68%	0.77%
HPD_05	0.400 V	1.916 mA·cm^−2^	0.015 mW	32.57%	0.25%

**Table 3:** Maximum and measured voltages at different frequencies and distances for the fabricated HPDs.

Devices	V_max_. 1 kHz, 0 cm	V_50_. 50 kHz, 0 cm	V_1_. 1 kHz, 10 cm	V_50_. 50 kHz, 10 cm
REF	132.51 mV	44.17 mV	10.96 mV	7.88 mV
HPD_01	206.81 mV	90.74 mV	19.86 mV	11.30 mV
HPD_02	208.86 mV	71.22 mV	19.17 mV	11.98 mV
HPD_03	187.63 mV	56.49 mV	16.43 mV	11.30 mV
HPD_04	107.85 mV	39.38 mV	11.30 mV	13.70 mV
HPD_05	104.77 mV	34.92 mV	11.30 mV	13.70 mV

**Table 4:** Comparative values of the main electrical, optical, and performance parameters for the organic photodetectors (REF–HPD_05).

Devices	R_Shut_ [kΩ]	I_Johnson_ [A·Hz^−1/2^]	R (V ≈ 0 V) [A/W]	R (V ≈ −0.98 V) [A/W]	D* (V ≈ 0 V) [Jones]	D* (V ≈ −0.98 V) [Jones]	NEP (V ≈ 0 V) [W·Hz^−1/2^]	NEP (V ≈ −0.98 V) [W·Hz^−1/2^]	V_max_. 1 kHz, 0 cm [mV]	V_50_. 50 kHz, 10 cm [mV]
REF	372	2.11 × 10^−13^	0.0343	0.0581	3.46 × 10^10^	1.32 × 10^10^	7.08 × 10^−12^	1.85 × 10^−11^	132.51	7.88
HPD_01	93	4.21 × 10^−13^	0.0825	0.1138	4.69 × 10^10^	1.41 × 10^10^	5.22 × 10^−12^	1.74 × 10^−11^	206.81	11.30
HPD_02	87	4.37 × 10^−13^	0.0775	0.1116	3.95 × 10^10^	1.26 × 10^10^	6.21 × 10^−12^	1.94 × 10^−11^	208.86	11.98
HPD_03	121	3.70 × 10^−13^	0.0599	0.0980	3.70 × 10^10^	1.18 × 10^10^	6.62 × 10^−12^	2.07 × 10^−11^	187.63	11.30
HPD_04	149	3.33 × 10^−13^	0.0515	0.0889	3.57 × 10^10^	1.24 × 10^10^	6.86 × 10^−12^	1.94 × 10^−11^	107.85	13.70
HPD_05	255	2.55 × 10^−13^	0.0192	0.0445	1.45 × 10^10^	7.48 × 10^9^	1.69 × 10^−11^	3.28 × 10^−11^	104.77	13.70

Specific detectivity (D*).

**Table 5:** Performance comparison between the optimized device (HPD_01) and the nanoparticle-free reference (REF).

Parameter	REF	HPD_01	Improvement
J_SC_ (mA·cm^2^)	3.45	8.21	+138%
V_OC_ (V)	0.478	0.401	−16%
FF (%)	35.4	42.4	+20%
PCE (%)	0.58	1.39	+140%
R (V ≈ 0 V (A·W^−1^))	0.034	0.083	+141%
R (V ≈ −0.98 V (A·W^−1^))	0.058	0.114	+96%
D* (V ≈ 0 V (Jones))	3.46 × 10^10^	4.69 × 10^10^	+36%
NEP (V ≈ 0 V (W·Hz^−1^))	7.08 × 10^−12^	5.22 × 10^−12^	−26%
f (−3 dB)	8.4	17.0	+102%
V_max_ 1 kHz, 0 cm (mV)	132.5	206.8	+56%
V_max_ 50 kHz, 10 cm (mV)	7.88	11.3	+43%

Specific detectivity (D*).

**Table 6:** Comparison of key figures of merit with representative literature values.

Reference	Active Layer	Structure	R (A/W)	D* (Jones)	f (−3 dB)	VLC	Bias (V)
[Arredondo_2013] [64]	P3HT:PCBM	Direct	0.18	--	790 kHz	Audio signal, indoors	−6
[Salamandra_2020] [15]	P3HT:PCBM	Inverted	--	--	~1 MHz	BER < 10^−9^ at 1 MHz	Reverse
[Kim_2024] [65]	P3HT:PCBM + CsPbBr_3_ NCs	PM-OPD	--	High D*	~0.5 kHz	--	Reverse
This work—REF	P3HT:PCBM	Direct	0.034	3.46 × 10^10^	8.4 kHz	Up to 1 MHz, >15 cm	0
This work—HPD_01	P3HT:PCBM + 2.06 wt% CsPbBr_3_	Direct	0.083	4.69 × 10^10^	17.0 kHz	Up to 1 MHz, >15 cm	0

## Data Availability

The raw data supporting the conclusions of this article will be made available by the authors on request.
